# Four-week inhibition of the renin–angiotensin system in spontaneously hypertensive rats results in persistently lower blood pressure with reduced kidney renin and changes in expression of relevant gene networks

**DOI:** 10.1093/cvr/cvae053

**Published:** 2024-03-19

**Authors:** Sean G Byars, Priscilla R Prestes, Varaporn Suphapimol, Fumihiko Takeuchi, Nathan De Vries, Michelle C Maier, Mariana Melo, David Balding, Nilesh Samani, Andrew M Allen, Norihiro Kato, Jennifer L Wilkinson-Berka, Fadi Charchar, Stephen B Harrap

**Affiliations:** The Florey Institute of Neuroscience and Mental Health, University of Melbourne, Parkville, Victoria, Australia; Health Innovation and Transformation Centre, Federation University, Ballarat, Victoria, Australia; Department of Anatomy & Physiology, School of Biomedical Sciences, University of Melbourne, Parkville, Victoria 3010, Australia; Department of Gene Diagnostics and Therapeutics, National Center for Global Health and Medicine, Tokyo, Japan; Health Innovation and Transformation Centre, Federation University, Ballarat, Victoria, Australia; Health Innovation and Transformation Centre, Federation University, Ballarat, Victoria, Australia; Department of Anatomy & Physiology, School of Biomedical Sciences, University of Melbourne, Parkville, Victoria 3010, Australia; Melbourne Integrative Genomic and School of Mathematics & Statistics, University of Melbourne, Victoria, Australia; Department of Cardiovascular Sciences, University of Leicester, Leicester, UK; The Florey Institute of Neuroscience and Mental Health, University of Melbourne, Parkville, Victoria, Australia; Department of Gene Diagnostics and Therapeutics, National Center for Global Health and Medicine, Tokyo, Japan; Department of Anatomy & Physiology, School of Biomedical Sciences, University of Melbourne, Parkville, Victoria 3010, Australia; Health Innovation and Transformation Centre, Federation University, Ballarat, Victoria, Australia; Department of Anatomy & Physiology, School of Biomedical Sciences, University of Melbourne, Parkville, Victoria 3010, Australia

**Keywords:** Hypertension, Prevention, SHR, Renin

## Abstract

**Aims:**

Prevention of human hypertension is an important challenge and has been achieved in experimental models. Brief treatment with renin–angiotensin system (RAS) inhibitors permanently reduces the genetic hypertension of the spontaneously hypertensive rat (SHR). The kidney is involved in this fascinating phenomenon, but relevant changes in gene expression are unknown.

**Methods and results:**

In SHR, we studied the effect of treatment between 10 and 14 weeks of age with the angiotensin receptor blocker, losartan, or the angiotensin-converting enzyme inhibitor, perindopril [with controls for non-specific effects of lowering blood pressure (BP)], on differential RNA expression, DNA methylation, and renin immunolabelling in the kidney at 20 weeks of age. RNA sequencing revealed a six-fold increase in renin gene (*Ren*) expression during losartan treatment (*P* < 0.0001). Six weeks after losartan, arterial pressure remained lower (*P* = 0.006), yet kidney *Ren* showed reduced expression by 23% after losartan (*P* = 0.03) and by 43% after perindopril (*P* = 1.4 × 10^−6^) associated with increased DNA methylation (*P* = 0.04). Immunolabelling confirmed reduced cortical renin after earlier RAS blockade (*P* = 0.002). RNA sequencing identified differential expression of mRNAs, miRNAs, and lncRNAs with evidence of networking and co-regulation. These included 13 candidate genes (*Grhl1*, *Ammecr1l*, *Hs6st1*, *Nfil3*, *Fam221a*, *Lmo4*, *Adamts1*, *Cish*, *Hif3a*, *Bcl6*, *Rad54l2*, *Adap1*, *Dok4*), the miRNA miR-145-3p, and the lncRNA AC115371. Gene ontogeny analyses revealed that these networks were enriched with genes relevant to BP, RAS, and the kidneys.

**Conclusion:**

Early RAS inhibition in SHR resets genetic pathways and networks resulting in a legacy of reduced *Ren* expression and BP persisting for a minimum of 6 weeks.

## Graphical Abstract

**Graphical Abstract:**
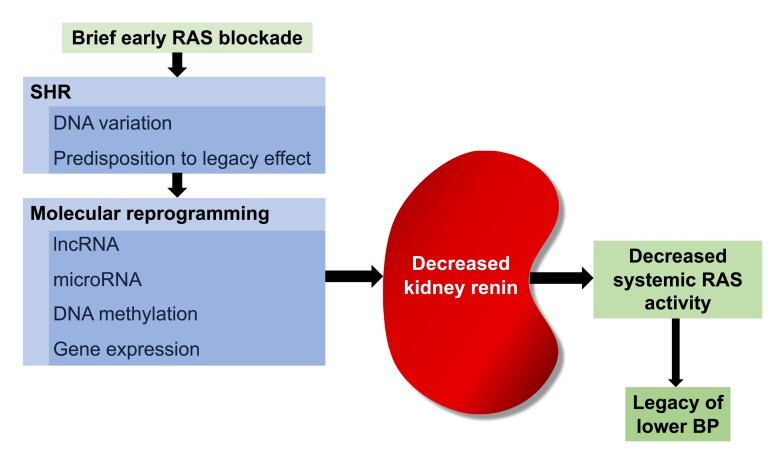

**Time of primary review: 32 days**

**See the editorial comment for this article ‘The epigenetic legacy of renin–angiotensin system inhibition in preventing hypertension’, by R. Nosalski and M. Lemoli, https://doi.org/10.1093/cvr/cvae076.**

## 1. Introduction

The ability to prevent the development of hypertension by treatment in early life would be an important advance in human health.^1^ This possibility has been raised by extensive research in the spontaneously hypertensive rat (SHR) model of human hypertension in which the blood pressure (BP) rises during adolescence to hypertensive levels in adulthood. This process is driven by an inherited genetic hypertensive ‘programme’. Numerous independent studies have shown that administration of inhibitors of the renin–angiotensin system (RAS) such as angiotensin-converting enzyme inhibitors (ACEi)^2,3^ or angiotensin receptor blockers (ARB)^4^ in the prehypertensive period results in a permanent reduction in SHR BP and increased lifespan.^5^ Our hypothesis is that the early RAS inhibition ‘reprogrammes’ the renal expression of genetic factors leading to hypertension in SHR.

RAS-inhibiting drugs are common and effective treatments for established human hypertension, but their potential for preventing hypertension is less clear. Three human trials have attempted to emulate the SHR prevention paradigm but with limited success.^1^ A more detailed understanding of the molecular basis of the legacy phenomenon in SHR should provide important clues for effective prevention of hypertension in humans.

The effects following RAS inhibition in SHR are not observed following treatment with other agents such as vasodilators (e.g. hydralazine), diuretics, alpha-adrenergic blockers, or calcium antagonists.^6–12^ This suggests the RAS is involved in both the genetic origins and prevention of SHR hypertension. Also, the legacy phenomenon is observed in some other hypertensive strains^7,13–15^ but not all,^16,17^ suggesting a genetic predisposition to the legacy phenomenon.

Here, we test our hypothesis that early RAS inhibition genetically reprogrammes the kidney in SHR. The prehypertensive SHR has reduced renal blood flow (RBF) and glomerular filtration rate (GFR) and high levels of kidney reninm^18,19^ and renal abnormalities have been linked to the inheritance of BP in cross-breeding experiments.^20^ The early renal haemodynamic abnormalities in SHR are reversed acutely by RAS inhibition^21^ and ameliorated in the long-term after early RAS inhibition.^2^ Renal transplantation between previously RAS-inhibited and untreated SHR causes BP to ‘follow the kidney’ such that kidneys from treated SHR will lower BP in untreated animals.^22^

We addressed the following specific questions in this study: (1) What are the long-term effects on RAS gene expression following early RAS inhibition? (2) What other changes in coding and non-coding gene expression^23^ are associated with long-term effects? (3) Is there evidence of differential DNA methylation that might explain changes in expression? (4) Do differentially expressed genes show patterns of co-regulation? (5) Are there identifiable clusters of differentially expressed genes relevant to biological processes, in particular BP? (6) Is there evidence of a class effect for RAS inhibitors? (7) Are there genomic sequence variants in SHR that might predispose to long-term changes in RNA expression?

## 2. Methods

### 2.1 Experimental outline

**Figure 1:**
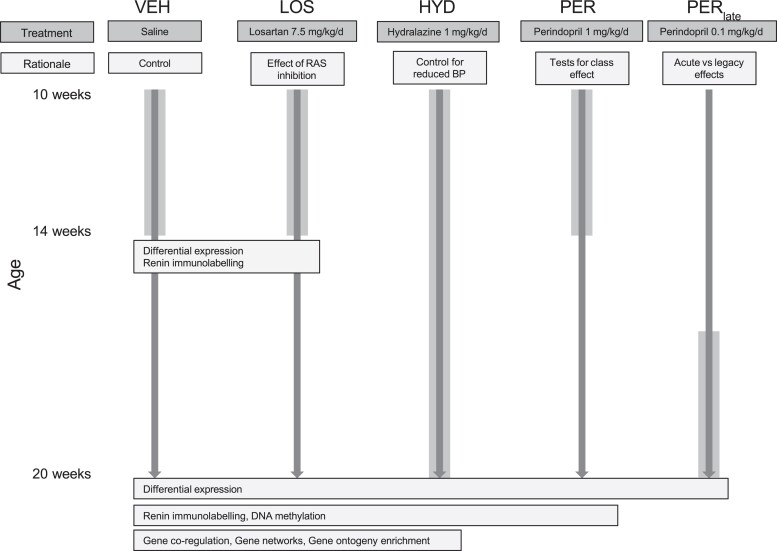
Outline of the basic experimental design showing the five experimental groups, their treatments, and experimental rationale. Treatment periods are shown by the wide grey vertical bars against age. The data obtained are shown in boxes at 14 and 20 weeks of age, spanning the groups analysed.

The basic experimental paradigm (*Figure 1*) involved treatment of male SHR from 10 to 14 weeks of age with cardiovascular measurements and molecular and histological analyses of renal cortices at 14 and 20 weeks of age. All experiments were approved by the University of Melbourne Animal Ethics Committee (ethics number 1313035) and were conducted in accordance with the NIH Guidelines for the Care and Use of Laboratory Animals. We obtained 6-week-old inbred male SHR from the Animal Resources Centre (Canning Vale, Western Australia). All treatments were delivered via minipumps (Alzet model 2004, Durect Corp., Cupertino, California).

#### 2.1.1 Losartan experiments

A primary set of experiments was based on a group of SHR treated with losartan from 10 to 14 weeks of age (LOS, 7.5 mg/kg/d, *n* = 18). Two control groups were used for identifying differential RNA expression. Our initial comparisons were made with vehicle (saline) treated SHR from 10 to 14 weeks of age (VEH, *n* = 18) to detect overall differences resulting from losartan treatment. We utilized a second control group to account for differences in LOS that might be the result of non-specific effects BP reduction *per se*. For this purpose, we included a group of SHR that were actively treated with the vasodilator hydralazine from 10 to 20 weeks of age (HYD, 1.0 mg/kg/d, *n* = 6) to mimic the BP effects following RAS inhibition (*Figure 1*). Differential expression (DE) analyses focused specifically on genes associated with the RAS and genome-wide analyses to identify candidate RNA species most strongly associated with the long-term effects following losartan at 20 weeks. These experiments provided detailed information regarding differential total mRNA and specific miRNA expression, DNA methylation, and the presence and nature of gene co-regulation, gene networks, and gene ontology enrichment.

#### 2.1.2 Perindopril experiments

We undertook additional experiments with the ACEi perindopril from 10 to 14 weeks of age (PER, 1.0 mg/kg/d, *n* = 11) to test specific hypotheses generated in the losartan experiments for evidence of a class effect of early RAS inhibition. Finally, to compare effects of active RAS inhibition vs. the legacy of early treatment, we included a group of SHR (PER_late_) that was treated with a lower dose of perindopril (0.1 mg/kg/d, *n* = 4) from 16 to 20 weeks of age to achieve a BP approximating the legacy effect following early RAS inhibition.

### 2.2 BP measurement and relative cardiac mass

Arterial pressures were measured using radiotelemetry between 10 and 20 weeks of age in a sample from four groups (VEH *n* = 2, LOS *n* = 2, HYD *n* = 2, PER = 3). These animals were excluded from sequencing analyses in case of confounding effects of surgery and *in situ* telemetry probes. Telemeters were inserted into the abdominal aorta at age 7 weeks (see Supplementary material online, *Methods*). Between 10 and 20 weeks of age, recordings were made over one 24 h period every week, from which we obtained the average daylight, resting 12 h measurement of mean arterial pressure (MAP) for each rat for that week. We also measured tail cuff systolic BP (SBP) in larger groups of animals (see Supplementary material online, *Methods*). In addition, we used relative cardiac mass (RCM) as an indication of the average BP. At the end of experiments, hearts were removed, blotted dry, and weighed, and RCM was calculated as the ratio of heart to body weight.

### 2.3 Kidney cortex collection

Animals were rapidly euthanized using 2% isoflurane and ketamine (100 mg/kg, i.m.; Lyppard, Dingley, Australia), and one kidney from each animal was dissected on ice to obtain cortical tissue for RNA extraction as described in Supplementary material online, *Methods*. The other kidney was immediately fixed in formalin for histological studies.

### 2.4 RNA sequencing and methylation analyses

Sequencing was performed on individual kidney cortex samples. Total RNA sequencing to capture coding and non-coding genes (e.g. mRNA, lncRNA, snoRNA), miRNA, and methylation sequencing was performed by the Australian Genome Research Facility (AGRF, Melbourne, Australia). Quality control of total RNA sequencing data and miRNA sequencing data with FastQC revealed high-quality sequence and base scores (see Supplementary material online, *Methods*). Total RNA sequencing was also obtained from Novogene (Beijing, China) to permit confirmation of VEH and LOS DE results. A total of three sequencing runs by AGRF and Novogene were made to accommodate all samples, and DE analyses were made only by comparison within individual runs to avoid potential batch effects. Differences in expression are presented as log_2_ fold changes (log_2_FC).

All total RNA sequencing utilized the same parameters including rRNA removal (Ribo-Zero depletion) and Illumina HiSeq 150 bp paired-end sequencing at high depth (∼100 million reads per sample). Illumina NovaSeq (50 bp single-end reads, ∼10–20 million reads per sample) was used for miRNA sequencing. Illumina HiSeq (100 bp single-end reads, 30 million reads per sample) was used for methylation sequencing using the reduced representation bisulphite sequencing (RRBS) technique. Details regarding read quality, alignment and quantification, data normalization, and analyses of DE are provided in Supplementary material online, *Methods*.

### 2.5 Gene co-regulation, gene network, and gene ontogeny enrichment analyses

To explore potential gene co-regulation, gene networks, and enriched gene ontology terms related to LOS treatment, we undertook a complimentary series of analyses, the details for which are provided in Supplementary material online, *Methods*.

First, we investigated co-regulatory relationships between differentially expressed protein-coding and non-coding RNA species to explore possible changes in regulatory machinery following losartan treatment. We employed expression data from the total RNA (see Supplementary material online, *Table S1*) and miRNA (see Supplementary material online, *Table S2*) data sets in multivariate analyses using regularized canonical correlation analysis (rCCA) and other tools in the R package mixOmics version 6.6.2.^24^

Next, we used weighted correlation network analysis (WGCNA version 1.68^25^) to identify networks comprising distinct clusters of co-expressed genes (modules) related to the long-term effects of LOS treatment. WGCNA networks were generated separately for mRNA, small-RNAs, and miRNA. Expression data from genes related to LOS treatment was used to build these networks (see Supplementary material online, *Tables S3*–*S5*) and selected so that leading differentially expressed genes were prioritized while also including sufficient numbers to allow network construction (see Supplementary material online, *Methods*). Within each gene module, hub genes that were central to gene networks were identified.^24^ Finally, we correlated module eigengenes (ME) with BP. An ME represents each module's summary expression profile and is a useful tool for studying relationships between different modules and relevant physiological phenotypes. To obtain insights into biological pathways that may be altered in response to treatment, we undertook gene ontology enrichment analyses (see Supplementary material online, *Methods*).

### 2.6 Renin immunolabelling

Immunohistochemistry for renin was performed as described previously,^26,27^ and all analyses were blinded to the treatment groups. Briefly, rat kidneys were fixed in 10% buffered formalin. Five μm paraffin sections of kidney were incubated with a polyclonal mouse renin protein antibody raised against pure mouse submandibular gland renin (1:8000). A negative control without the primary antibody was included (see Supplementary material online, *Methods*). For quantitation, four randomly chosen sections from each kidney at least 125 μm apart were selected. Images of renin immunolabelling associated with the juxtaglomerular apparatus (JGA) from 50 glomeruli per section were captured, and the proportion of renin-positive JGAs per section was averaged across 4 sections from each sample.

### 2.7 Renin gene sequence variants

We searched for DNA variants in SHR-derived strains (SHR/SHRSP) that might predispose to the long-term antihypertensive effects following RAS inhibition. We used whole genome sequencing data variants in and around the renin gene for SHR/Utx, a substrain of University of Texas, Houston; SHRSP/Bbb and WKY/Bbb, substrains of Max Delbruck Center for Molecular Medicine;^28^ and SHRSP/Izm, SHR/Izm, and WKY/Izm, substrains of Japanese colony.^29^ We aligned the NGS reads to the BN reference (mRatBN7.2) for practical reasons in the interval of 44 781 715–44 808 987 on RNO13 and sought DNA variation [single nucleotide polymorphisms (SNPs), insertions, deletions, nucleotide repeats] in SHR and SHRSP that were not observed in WKY or BN sequences. Transcription factor (TF) binding sites were predicted using PROMO (https://alggen.lsi.upc.es/cgi-bin/promo_v3/promo/promoinit.cgi?dirDB=TF_8.3) with humans selected as factor’s species, considering the reported similarities of TF motifs between mammals.^30^

### 2.8 Statistical analyses

Parametric analyses [*t*-tests, analyses of variance (ANOVA), repeated measures ANOVA] were used to compare data that are close to normally distributed and presented as mean ± standard deviation (SD) unless otherwise specified. Non-parametric tests (Mann–Whitney test, Kruskal–Wallis test) were used for non-normal data that are presented as median and interquartile range (IQR) unless otherwise stated. Tests were performed using IBM SPSS Statistics version 27. Specialized statistical approaches are described in the relevant sections in Supplementary material online, *Methods*. The data underlying this article will be shared on reasonable request to the corresponding author.

## 3. Results

### 3.1 Losartan experiments

#### 3.1.1 Cardiovascular effects of treatment

**Figure 2:**
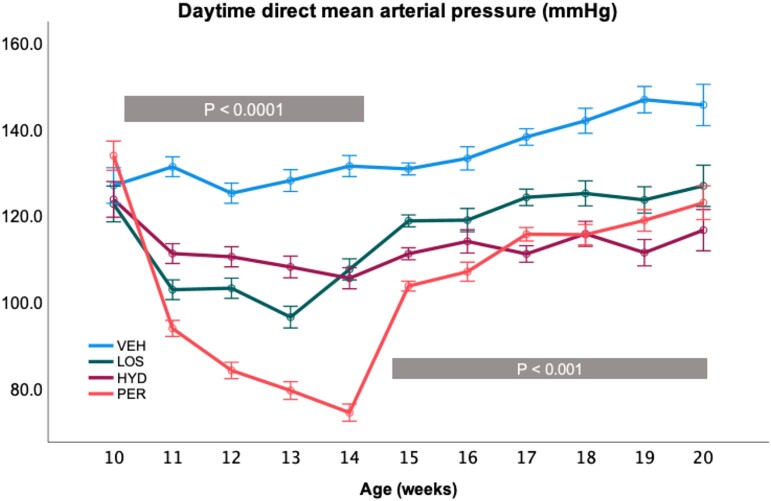
Direct MAP in male SHR from 10 to 20 weeks of age. Animals received saline (VEH, *n* = 2, blue), losartan (LOS, *n* = 2, green), or perindopril (PER, *n* = 3, orange) between 10 and 14 weeks of age or hydralazine (HYD, *n* = 2, purple) between 10 and 20 weeks of age. Weekly values are summarized as estimated marginal means from the repeated measures ANOVA, and error bars represent the SEM. The *P* values represent the overall effect of treatment during (10–14 weeks) and after treatment for LOS and PER (15–20 weeks) by repeated measures ANOVA. Individual treatment comparisons with VEH are given in the text.

The direct telemetric MAP recordings (*Figure 2*) demonstrated that losartan (*P* = 0.005 by repeated measures ANOVA) and hydralazine (*P* = 0.009) significantly reduced BP during treatment from 10 to 14 weeks of age. On cessation of treatment, the BP of losartan-treated SHR rose to levels similar to the SHR receiving ongoing hydralazine treatment and both remained significantly lower than control SHR (LOS *P* = 0.006; HYD *P* = 0.005). The tail cuff SBP recordings (mean ± SD) confirmed these differences at 14 weeks (VEH: *n* = 7, 206 ± 16 mmHg; LOS: *n* = 10, 145 ± 22 mmHg; HYD: *n* = 5, 153 ± 12 mmHg) (ANOVA *P* < 0.0001) and 20 weeks (VEH: *n* = 8, 220 ± 18 mmHg; LOS: *n* = 8, 187 ± 16 mmHg; HYD: *n* = 3, 153 ± 8 mmHg) (ANOVA *P* < 0.0001). At 14 weeks of age, mean RCM was significantly reduced in LOS (*n* = 6, 3.21 ± 0.14 g/kg) and HYD (*n* = 2, 3.46 ± 0.01 g/kg) compared with the VEH (*n* = 4, 3.63 ± 0.13 g/kg) (ANOVA *P* = 0.002) group. Similarly, at 20 weeks of age, RCM was significantly less in LOS (*n* = 11, 3.36 ± 0.12 g/kg) and HYD (*n* = 3, 3.34 ± 0.28 g/kg) compared with VEH (*n* = 8, 3.61 ± 0.12 g/kg) (ANOVA *P* = 0.001).

#### 3.1.2 RAS gene expression

During losartan treatment at 14 weeks, there were, as anticipated, significant changes in the expression of several genes involved in the RAS and its signalling pathways (see Supplementary material online, *Table S6*). The most prominent was a significant increase in renin gene (*Ren*) mRNA [log_2_FC = 2.46, false discovery rate (FDR) *q* = 1.3 × 10^−133^]. Other significant changes included increased expression of the genes encoding angiotensinogen (*Agt*: log_2_FC = 0.50, FDR *q* = 3.2 × 10^−5^), angiotensin II receptor–associated protein^31^ (*Agtrap*: log_2_FC = 0.27, FDR *q* = 2.2 × 10^−5^), and transforming growth factor beta 1^32^ (*Tgfb1*: log_2_FC = 0.38, FDR *q* = 2.1 × 10^−5^) and reduced expression of renin receptor ATPase H+ transporting accessory protein 2^33^ (*Atp6ap2*: log_2_FC = −0.22, FDR *q* = 4.3 × 10^−5^), mitogen-activated protein kinase 1^34^ (*Mapk1*: log_2_FC = −0.25, FDR *q* = 0.004), and SRY-box TF 6^35^ (*Sox6*: log_2_FC = −0.25, FDR *q* = 0.018).

At 20 weeks of age, *Ren* was the only RAS gene to show significant DE following prior losartan treatment, with expression reduced compared with both VEH (log_2_FC = −0.31, *P* = 9.5 × 10^−6^; Supplementary material online, *Table S7*) and HYD (log_2_FC = −0.37, *P* = 0.03; Supplementary material online, *Table S8*).

#### 3.1.3 Genome-wide DE analyses

Comparing the renal cortices from 20-week-old LOS and VEH SHR, we identified 164 differentially expressed genes (see Supplementary material online, *Table S7*) sequenced by AGRF and 177 differentially expressed genes in the Novogene sequencing results from the same samples (see Supplementary material online, *Table S9*). There were 35 genes that showed significant (FDR *q* < 0.05) and consistent (in terms of direction and magnitude) DE in both the AGRF and Novogene analyses (see Supplementary material online, *Figure S1*), and we designated these genes as ‘validated’ (*Table 1*).

**Table 1:** Genes showing DE in kidney cortices at 20 weeks of age after prior treatment with losartan

		DE				WGCNA			DME
		LOS vs. VEH		LOS vs. HYD					
	Chr	Log_2_FC	FDR *q*	Log_2_FC	FDR *q*	Module	SBP (*r*)	Hub gene	Log_2_FC
**A. Candidate genes**									
Adamts1	11	−0.73	<0.001	−0.58	0.003	Purple		Yes	0.3
Adap1	12	−0.45	<0.001	−0.59	<0.001	Salmon		Yes	0.37
Bcl6	11	1.47	<0.001	1.12	<0.001	Mediumpurple3			
Cish	8	−2.43	<0.001	−1.97	<0.001	Darkmagenta			
Fam221a	4	−0.72	<0.001	−0.52	0.001	Purple		Yes	
Grhl1	6	−1.21	<0.001	−0.85	<0.001	Purple		Yes	
Lmo4	2	−0.52	<0.001	−0.53	<0.001	Purple		Yes	
Hs6st1	9	−0.59	<0.001	−0.31	0.041	Purple		Yes	
Rad54l2	8	−0.37	<0.001	−0.38	0.014	Plum1		Yes	
Dok4	19	−0.36	0.007	−0.3	0.032	Salmon		Yes	
Nfil3	17	−0.73	0.014	−0.73	0.001	Purple		Yes	0.28
Hif3a	1	0.8	0.017	1.25	<0.001	Mediumpurple3			
Ammecr1l	18	−0.27	0.03	−0.3	0.01	Purple		Yes	
**B**									
Rspry1	19	−0.36	<0.001	−0.26	0.053	Purple		Yes	
Ipmk	20	−0.48	<0.001	−0.31	0.065	Purple		Yes	0.46
Nrg1	16	−0.51	<0.001	−0.37	0.203	Plum1		Yes	
Adm2	7	−0.45	0.001	−0.25	0.09	Purple		Yes	
Znf750	10	0.47	0.002	0.33	0.051	Black			0.5
Tfap4	10	0.43	0.002	0.33	0.118	Yellowgreen		Yes	
Irak2	4	−0.38	0.002	−0.2	0.206	Purple		Yes	
Csrnp3	3	−0.5	0.003	−0.36	0.129	Purple		Yes	0.57
AABR07063425.3	6	0.62	0.003	0.49	0.286	Brown		Yes	1.35
Syde2	2	0.35	0.005	0.23	0.056	Yellowgreen		Yes	
Pik3cd	5	−0.31	0.017	−0.33	0.105	Salmon			
AABR07008066.2	2	0.41	0.015	0.36	0.069	Darkred	−0.63	Yes	
Bcl2l11	3	0.36	0.02	0.33	0.095	Darkred	−0.63		
Hlx	13	−0.5	0.034	−0.36	0.062	Salmon		Yes	
**C**									
Prima1	6	0.56	0.008	−0.59	0.001	Lightsteelblue1	−0.71		
Nuak2	13	0.38	<0.001	0.1	0.809	White		Yes	
Svopl	4	0.8	<0.001	0.09	0.862	Lightcyan		Yes	
Errfi1	5	−0.79	0.003	−0.22	0.663	Lightgreen			
Ly75	3	−0.47	0.015	−0.03	0.971	Green	0.75		
Ndel1	10	−0.26	0.028	−0.14	0.402	Saddlebrown			
Nfam1	7	−0.43	0.031	−0.2	0.558	Green	0.75	Yes	
Smad7	18	0.21	0.047	−0.05	0.853	Lightsteelblue1	−0.71		

These 35 ‘validated’ genes showed DE as log_2_FC between LOS and VEH in two independent RNA sequencing analyses (AGRF and Novogene). Differences were considered significant when FDR *q* < 0.05. They are grouped as A, candidate genes that also showed significant (FDR *q* < 0.05) DE compared with HYD; B, genes that showed nominally significant (*P* < 0.05) DE compared with HYD; and C, genes that did not show DE when compared with HYD. WGCNA gene network statistics include module assignment, significant correlations (*r*) of ME with SBP, and hub gene designation. Differential methylation (DME) fold change values are provided for genes that contained significantly differentially methylated CpG clusters—for genes with more than one DME CpG cluster, only the leading (most significant) fold change value is provided. Chr, chromosome.

Using HYD as controls (see Supplementary material online, *Table S8*), we identified 13 genes among the 35 validated genes that showed significant DE in the same direction as VEH analyses when compared with HYD (*Table 1A*). As their expression could not be ascribed to lower BP *per se*, we nominated these 13 loci as ‘candidates’ for the long-term effects of losartan: *Grhl1*, *Ammecr1l*, *Hs6st1*, *Nfil3*, *Fam221a*, *Lmo4*, *Adamts1*, *Cish*, *Hif3a*, *Bcl6*, *Rad54l2*, *Adap1*, and *Dok4* (see *Table 1A*). Another 14 genes showed significant DE against VEH but only nominal differences with HYD (*Table 1B*).

**Figure 3:**
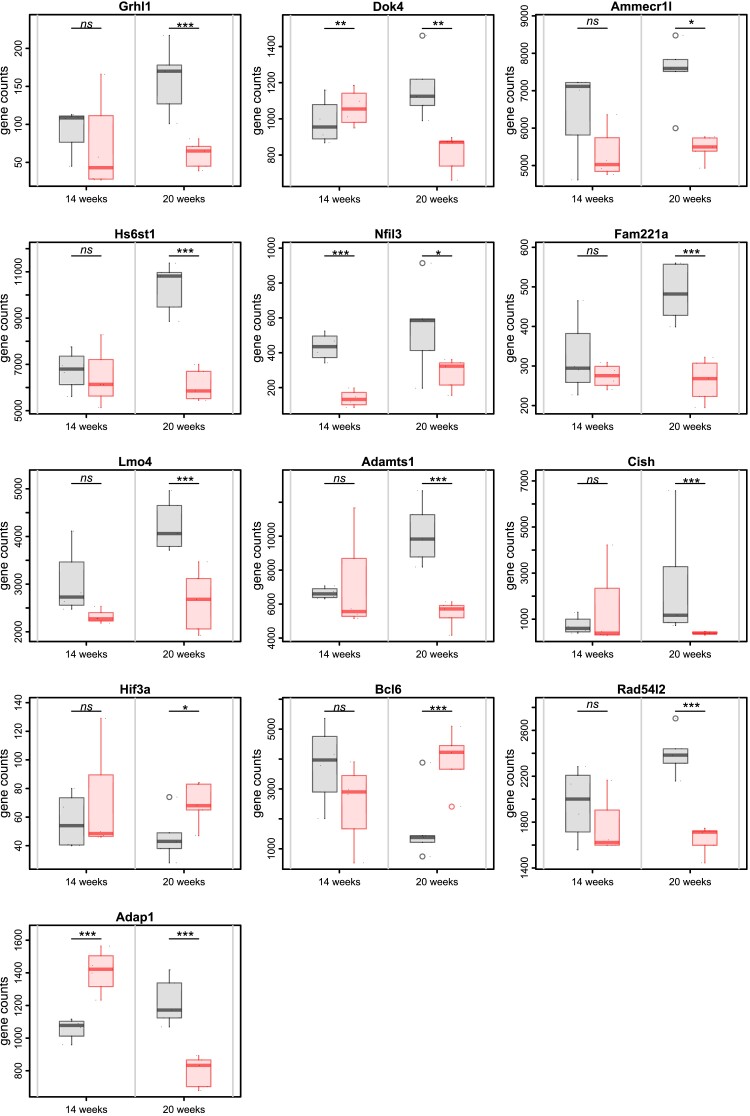
Changes in expression of 13 candidate genes at 14 and 20 weeks in losartan and control SHR animals. Gene expression (counts) is displayed individually (black points) and summarized as boxplots (vehicle, grey; losartan, red). FDR *q*-values (***<0.001; **<0.01; *<0.05; ns > 0.05) are from differential gene expression analyses.

The expression patterns of these 13 candidates at 14 and 20 weeks are shown in *Figure 3*. Although all candidates showed significant DE at 20 weeks, only three candidates: *Nfil3* (log_2_FC = −1.54, FDR *q* = 6.6 × 10^−13^), *Adap1* (log_2_FC = 0.53, FDR *q* = 2.8 × 10^−6^), and *Dok4* (log_2_FC = 0.25, FDR *q* = 0.007), were differentially expressed during active losartan treatment at 14 weeks, suggesting they are sensitive to the effects of losartan itself.

In addition, we identified 45 miRNAs that showed DE between the LOS and VEH groups at 20 weeks (*Table 2*). Most of these miRNAs (*n* = 33) showed up-regulation in the LOS animals compared with controls.

**Table 2:** miRNAs showing DE at 20 weeks in response to losartan treatment in the spontaneously hypertensive rat

		LOS vs. VEH		WGCNA		
	Chr	Log_2_FC	FDR *q*	Module	SBP (*r*)	Hub gene
**miR-340-5p**	10	1.47	0.043	Turquoise		Yes
**miR-872-5p**	5	1.29	0.038	Turquoise		Yes
**miR-141-3p**	4	1.61	0.038	Turquoise		Yes
**miR-26b-5p**	9	1.11	0.038	Turquoise		Yes
**miR-21-5p**	10	1.47	0.038	Turquoise		Yes
**miR-101b-3p**	1	1.69	0.034	Turquoise		Yes
**miR-450a-5p**	X	1.41	0.033	Turquoise		Yes
**miR-542-3p**	X	1.96	0.032	Turquoise		Yes
**miR-499-5p**	3	1.60	0.032	Turquoise		Yes
**miR-7a-5p**	17	1.52	0.032	Turquoise		Yes
**miR-101a-3p**	5	2.02	0.032	Turquoise		Yes
**miR-374-3p**	X	2.08	0.032	Turquoise		Yes
**miR-19a-3p**	15	2.55	0.029	Turquoise		Yes
**miR-32-5p**	5	3.18	0.025	Turquoise		Yes
**miR-379-5p**	6	0.80	0.045	Turquoise		
**miR-7b**	9	1.57	0.044	Turquoise		
**miR-376c-3p**	6	1.60	0.043	Turquoise		
**miR-190b-5p**	2	2.20	0.043	Turquoise		
**miR-33-5p**	7	3.78	0.043	Turquoise		
**miR-547-3p**	X	1.78	0.043	Turquoise		
**miR-489-5p**	4	2.03	0.039	Turquoise		
**miR-142-3p**	10	1.86	0.032	Turquoise		
**let-7i-5p**	7	0.88	0.032	Turquoise		
**let-7g-5p**	8	1.10	0.032	Turquoise		
**miR-3559-5p**	X	1.75	0.026	Turquoise		
**miR-1b**	3	1.78	0.026	Turquoise		
**miR-190a-5p**	8	2.94	0.026	Turquoise		
**miR-144-3p**	10	4.47	0.017	Turquoise		
**miR-345-5p**	6	−0.71	0.032	Red		Yes
**miR-326-3p**	1	−0.82	0.029	Red		Yes
**miR-145-5p**	18	−0.99	0.016	Red		Yes
**miR-3547**	10	−1.45	0.044	Red		
**miR-145-3p**	18	0.88	0.034	Yellow		Yes
**miR-181b-1-3p**	13	1.84	0.032	Yellow		
**miR-98-5p**	X	1.17	0.026	Yellow		
**miR-185-5p**	11	0.59	0.026	Yellow		
**let-7f-5p**	17	1.25	0.025	Yellow		
**miR-423-5p**	10	−0.79	0.038	Brown		Yes
**miR-339-5p**	12	−0.86	0.026	Brown		Yes
**miR-664-3p**	18	−0.82	0.032	Brown		
**miR-193b-3p**	14	−0.96	0.032	Brown		
**miR-15b-5p**	2	−0.71	0.038	Green		Yes
**miR-342-3p**	6	−0.74	0.043	Blue		
**miR-365-3p**	10	−0.72	0.040	Blue		
**miR-150-5p**	1	−0.81	0.038	Blue		

WGCNA gene network statistics include module assignment, significant correlations (*r*) of ME with SBP, and hub gene designation. Chr, chromosome

#### 3.1.4 Correlations between coding and non-coding RNA

**Figure 4:**
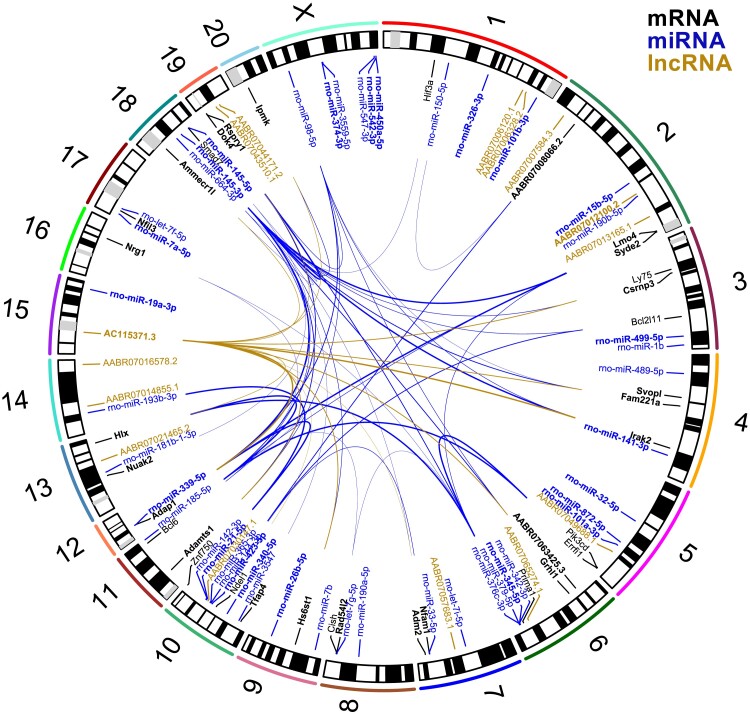
Leading differentially expressed genes and their associations. Including 35 mRNAs (black text) from *Table 1*, 45 miRNAs (blue text) from *Table 2*, and 15 lncRNAs (gold text). Hub genes identified from the WGCNA analysis are in bold text. For each of the 35 mRNAs, the leading 2 negative correlations < −0.8 (if present) with non-coding genes are shown as thicker lines representing larger negative associations (see Supplementary material online, *Figure S2* and *Table S10*).

Given the potential control of gene expression by non-coding RNA, we investigated multivariate correlations between the differentially expressed coding and non-coding RNAs. Those which are highly correlated (|*r*| ≥ 0.8) are shown in Supplementary material online, *Figure S2*. When considering large negative correlations (*r* < −0.8) between the 13 candidate genes and non-coding RNAs (*Figure 4*; Supplementary material online, *Table S10*), we found that the miRNA miR-145-3p was highly negatively correlated with 9 of the 13 candidates: *Adamts1*, *Hs6st1*, *Fam221a*, *Grhl1*, *Ammecr1l*, *Adap1*, *Dok4*, *Lmo4*, and *Rad54l2* (see Supplementary material online, *Table S10* and *Figure S2C*). We also noted that among the lncRNAs, AC115371^36^ was very highly correlated with numerous miRNAs (*n* = 29 of 45) and showed the highest correlation (*r* = 0.97) with miR-145-3p (see Supplementary material online, *Table S11* and *Figure S3*).

#### 3.1.5 Gene network analyses, modules, and SBP correlations

The WGCNA network analyses identified a total of 43 modules among 2844 protein-coding genes (see Supplementary material online, *Table S3* and *Figure S4A*) ranging in size from 25 to 207 genes each. Among the 13 candidate genes, 10 were identified as hub genes (*Table 1A*), suggesting a central role within these gene co-expression modules related to the legacy of losartan treatment. Seven of these (*Grhl1*, *Ammecr1l*, *Hs6st1*, *Nfil3*, *Fam221a*, *Lmo4*, *Adamts1*) were in the module purple (see Supplementary material online, *Table S3*). The genes *Adap1* and *Dok4* were in module salmon, and interestingly, these two genes had shown increased expression during losartan treatment at 14 weeks but significantly reduced expression at 20 weeks. The non-coding RNA species from the total RNA sequencing (*n* = 186) were allocated separately to 19 modules (see Supplementary material online, *Table S4* and *Figure S4B*), with miRNAs, snoRNAs, and lncRNAs (including AC115371.3) identified as hub genes. The miRNAs (*n* = 210) were grouped into seven modules (see Supplementary material online, *Table S5* and *Figure S4C*).

The correlation between SBP and the summary expression (eigengene) for each module was examined and found to be significantly correlated with nine mRNA modules, three non-coding RNA modules, and one miRNA module (see Supplementary material online, *Figure S4*).

#### 3.1.6 Gene ontogeny enrichment analysis

Differentially expressed genes related to 20-week LOS treatment showed significant enrichment (FDR *q* < 0.05) with 153 biological processes (see Supplementary material online, *Table S12B*). Given that many enrichment terms were very broad (i.e. including thousands of genes), we also explored nominally significant (*P* < 0.05) terms. We identified 10 terms that involved regulation of BP and/or RAS (see Supplementary material online, *Table S12A*) that were closely related (see Supplementary material online, *Table S12D*). Within these domains, there were two differentially expressed RAS-related genes (*Ren*, *Agt*) in nominally significant RAS-related pathways such as ‘regulation of blood volume by renin–angiotensin’ (GO:0002016, *P* = 0.002; Supplementary material online, *Table S12C*). There were also other differentially expressed genes (e.g. *Adm2*, *Bdkrb2*, *Edn3*, *Pde4d*) found in nominally significant parent terms such as ‘positive regulation of BP’ (GO:0045777, *P* = 0.003).

#### 3.1.7 Co-regulation of RAS gene expression

To explore potential co-regulation between RAS-specific and other differentially expressed genes in LOS animals, we investigated the multivariate correlation between *Ren* (and other relevant RAS genes) and the validated differentially expressed 35 mRNA and 45 miRNA at 20 weeks and with the 35 mRNA at 14 weeks during treatment (see Supplementary material online, *Tables S13*–*S15*, *Figure S5*, and *Methods*).

Among the RAS genes, *Ren* was most frequently correlated (*n* = 18 genes with |*r*| ≥ 0.8) with the 35 validated genes at 20 weeks (see Supplementary material online, *Table S13* and *Figure S5A*). This included 6 out of the 13 candidate genes, including the 2 strongest correlations (with *Bcl6* and *Hs6st1*). Only 1 other RAS gene (*Agt*) was highly correlated with 3 of the 35 validated genes at 20 weeks (see Supplementary material online, *Table S13*).

The opposite was apparent at 14 weeks (see Supplementary material online, *Figure S5B*). *Ren* (despite having markedly elevated expression) was less highly correlated (*n* = 6 genes with |*r*| ≥ 0.8) with the 35 validated genes, and only 3 of these (e.g. *Nfil3*, *Dok4* and *Adap1*) were among the 13 candidate genes (see Supplementary material online, *Table S14*). Many of the other RAS genes (*Agt*, *Agtrap*, *Atp6ap2*, *Mapk1*) that were not highly correlated at 20 weeks, however, were so at 14 weeks.

In terms of correlations between miRNA and RAS genes at 20 weeks of age (see Supplementary material online, *Table S15*), the highest correlation observed was between *Ren* and the miRNA miR-664-3p (see Supplementary material online, *Figure S5C*). Supplementary material online, *Figure S3*, shows the observed strong intercorrelations between *Ren*, miRNA, and lncRNA.

#### 3.1.8 Differential methylation

**Figure 5:**
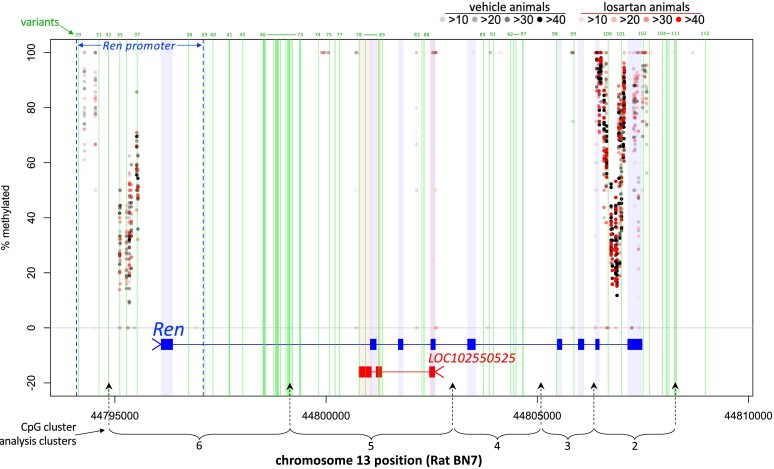
Patterns of methylation across *Ren* and LOC102550525. Detailed view of methylation % by vehicle and losartan animals (point shading reflects count number per CpG). Green vertical lines show position of variants (see variant description in Supplementary material online, *Table S18*) in and around *Ren*. General promoter region for *Ren* is indicated by dark blue dashed lines. Position of CpG clusters (2–6) across *Ren* is shown which corresponds to the cluster number in CpG cluster analysis (see Supplementary material online, *Table S16*).

Among the 13 candidate genes, the cluster-based analysis revealed significantly increased methylation at CpG clusters in 20-week LOS SHR for *Adamts1*, *Adap1*, and *Nfil3* (*Table 1A*; Supplementary material online, *Table S16*) corresponding with their lower RNA expression. Among the RAS genes, only *Ren* showed evidence of increased methylation in cluster 2 (see Supplementary material online, *Table S16*) that was localized in the 3′ end of *Ren* as shown in *Figure 5*. In analyses focused on gene promoter regions (see Supplementary material online, *Table S17*) among the 35 validated and RAS genes, only *Ren* showed significantly increased methylation (*Figure 5*; log_2_FC = 0.30, permutation *P*-value 0.04) at 20 weeks.

### 3.2 Perindopril experiments

#### 3.2.1 Cardiovascular effects of treatment

The direct MAP recordings in PER SHR are shown in *Figure 2* with significant reductions in pressure during (*P* = 0.0003 by repeated measures ANOVA) and after (*P* = 0.004) treatment. Compared with VEH, mean tail cuff SBP in the PER group was significantly reduced during (PER: *n* = 4, 125 ± 15 mmHg, vs. VEH: *n* = 4, 215 ± 10 mmHg, *t*-test *P* < 0.0001) and after (PER: *n* = 10, 161 ± 17 mmHg, vs. VEH: *n* = 8, 220 ± 18 mmHg, *t*-test *P* < 0.0001) treatment. This pattern was mirrored by the lower RCM at 14 (PER: *n* = 4, 2.81 ± 0.09 g/kg, vs. VEH: *n* = 4, 3.63 ± 0.13 g/kg, *t*-test *P* = 0.0001) and 20 (PER: *n* = 10, 3.27 ± 0.15 g/kg, vs. VEH: *n* = 12, 3.65 ± 0.26 g/kg, *t*-test *P* = 0.0001) weeks of age.

#### 3.2.2 Kidney Ren expression

We found reduced kidney cortex *Ren* expression compared with HYD at 20 weeks PER (log_2_FC: −0.80, *P* = 1.4 × 10^−6^). Active treatment with perindopril (0.1 mg/kg/d) between 16 and 20 weeks of age resulted in reduced SBP (*n* = 4, 179 ± 12 mmHg, *P* = 0.002) and RCM (3.24 ± 0.16 g/kg, *P* = 0.001) similar to PER animals. However, in distinct contrast to the reduction in *Ren* expression in PER animals, the PER_late_ animals showed a 3-fold increase in *Ren* expression (log_2_FC: 1.57, *P* = 7.6 × 10^−49^).

#### 3.2.3 Candidate gene expression

Although among the 13 candidates identified in the losartan experiments, 9 showed DE after perindopril in the same direction as losartan, only 1 showed significant change in the perindopril experiments—*Nfil3*. *Nfil3* expression was reduced in the kidneys of PER (log_2_FC −1.09, FDR *q* = 1.4 × 10^−16^). In addition, active perindopril treatment at 20 weeks was also associated with a significant reduction in *Nifl3* expression (log_2_FC −0.79, *P* = 1.2 × 10^−8^, FDR *q* = 2.2 × 10^−6^), mirroring what had been observed at 14 weeks with losartan treatment.

### 3.3 Renin immunolabelling

**Figure 6:**
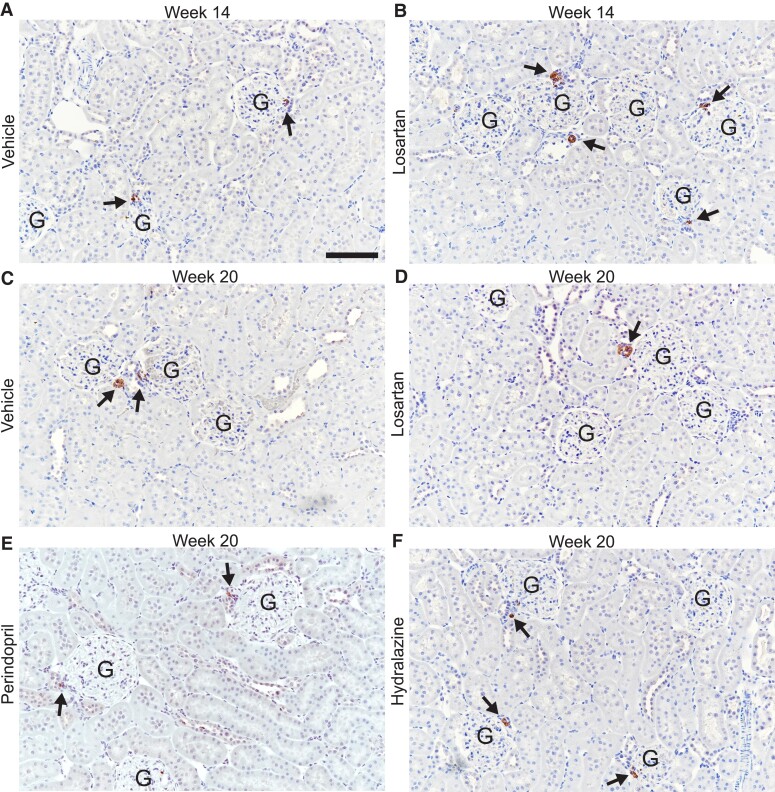
Renin immunolabelling in kidneys from 14-week (*A* and *B*) and 20-week-old (*C*–*F*) SHR treated with vehicle (*n* = 12), losartan (*n* = 13), perindopril (*n* = 8), and hydralazine (*n* = 6). Three μm paraffin sections counterstained with haematoxylin. G, glomerulus. Arrows denote renin immunolabelling (brown). Scale bar, 100 μm.

To complement the kidney *Ren* expression, we measured renin immunolabelling in the cortex of 14- and 20-week-old SHR (*Figure 6*). During treatment at 14 weeks, renin immunolabelling (summarized as median and IQR) was significantly higher in LOS SHR (*n* = 6, 45.2%, IQR 3.9%) compared with VEH (*n* = 4, 10.1%, IQR 6.5%) (*P* = 0.01 by Mann–Whitney test). However, at 20 weeks, renin immunolabelling was lower in LOS (*n* = 7, 7.1%, IQR 1.3%) and PER (*n* = 8, 10.8%, IQR 7.3%) kidneys compared with VEH (*n* = 8, 13.7%, IQR 5.8%), while renal renin was higher in HYD kidneys (*n* = 6, 18.3%, IQR 2.37%). Renin immunolabelling after any RAS inhibition (LOS and PER) (7.6%, IQR 5.1%) was lower than VEH (*P* = 0.05 by Mann–Whitney test) and HYD (*P* = 0.001 by Mann–Whitney test). These changes corroborated the *Ren* RNA expression results.

### 3.4 Renin gene sequence variants

The details of 112 variants identified in and around the SHR-derived strains (not observed in BN or WKY) are shown in Supplementary material online, *Table S18*. These included SNPs, nucleotide repeat variants, and insertion and deletions. Fifty-six of the variants were associated with changes in the sequence of putative TF binding sites including a previously reported variant^37^ in the first intron for GR-alpha. Several of the TFs already have established relationships with the control of renin expression and the RAS including FOXP3,^38^ RXR-alpha,^39^ PPAR-alpha,^39^ RBP-J,^40^ and c-Jun.^41^ Three sequence variants in the antisense lncRNA LOC102550525 represented changes to putative TFs binding sites (see Supplementary material online, *Table S18*).

## 4. Discussion

Since the initial observation some 40 years ago,^2^ numerous independent studies in SHR have established that brief RAS inhibition in younger animals can prevent the full development of genetic hypertension. Despite considerable effort, the explanation for this remarkable phenomenon has remained elusive. Here, we report for the first time that following early ARB or ACEi treatment in SHR, the persistent reduction in BP, instead of eliciting the expected reflex increase in renin,^42^ is associated with a reduction in kidney renin, as evidenced by significantly lower gene expression and protein immunolabelling.

The lower kidney renin is important to the legacy of early RAS inhibition in SHR because of the major role of the RAS in BP homeostasis and for the pathophysiology of SHR hypertension. Renin drives the RAS through the regulation of the initial and rate-limiting step of the RAS cascade. The kidney is the only organ capable of releasing enzymatically active renin.^43^ The kidneys and renin are relevant to the pathogenesis of SHR hypertension. High plasma and kidney renin activity^18,19^ and abnormalities of renal glomerular haemodynamics are seen during the development of SHR hypertension.^44^ Our previous cross-breeding studies have genetically linked BP, glomerular haemodynamics, and plasma renin activity to the development of hypertension.^20^ The involvement of kidney renin with the legacy effect also aligns with two other important observations. The first is that RAS inhibitors, but not other classes of antihypertensive drugs, produce a long-term reduction in BP. The second is that the kidneys are central to the legacy effect,^45^ as epitomized by transplantation in which the BP ‘follows the kidney’.

Surprisingly, the potential involvement of the RAS in long-term BP effects of RAS inhibition in SHR has received relatively little attention. Previous studies revealed long-term reductions in plasma angiotensinogen levels after early ACE inhibition^46^ and reduced renal angiotensin II and aldosterone,^4^ but kidney renin was not measured. However, in the closely related SHRSP strain, the development of severe renal damage (as a result of advance age^7^ or L-NAME treatment^12^) caused an increase in kidney *Ren* expression, but this response was significantly attenuated in SHRSP that had received earlier treatment with the ARB candesartan, suggesting long-term resetting of kidney renin.

Why might kidney renin remain low after early RAS inhibition in SHR? One physiological explanation could be reduced sympathetically mediated renin release.^43^ We have no direct measure of sympathetic activity, but the heart rate measured by telemetry in freely moving SHR treated with either losartan or perindopril was not different to controls either during or after treatment (data not shown). Furthermore, transplantation studies^22^ in which renal nerves are severed suggest that factors other than renal sympathetic activity explain the long-term lower BP.

Instead, we believe that the reduced kidney renin is the result of changes in gene networks by early RAS inhibition. Our genomic analyses provided insights into the nature of these changes. We found significant differences in expression of genes, non-coding RNA, and DNA methylation. Our network analyses revealed changes were strongly correlated with the reduced *Ren* expression. Furthermore, the gene ontology analyses indicated changes in expression of genes with known relationships with RAS, BP, and the kidneys. These networks included 13 candidate genes that were differentially expressed at 20 weeks of age after early RAS inhibition and that we believe are likely to cause or sustain the reduced *Ren* expression and the legacy of lower BP.

Among the 13 candidates, 6 (*Grhl1*, *Nfil3*, *Adamts1*, *Hs6st1*, *Adap1*, *Bcl6*) have established cardiovascular involvement and interactions with the RAS and 9 showed DE in the same direction after perindopril and losartan, possibly reflecting class effects of RAS inhibition. Within these candidates, *Nfil3* is of special interest. Only *Nfil3* showed significant evidence of reduced expression during (at 14 weeks in LOS and at 20 weeks in PER_late_) and after (20 weeks in LOS and PER) RAS inhibition treatment. There was also evidence for significantly increased methylation of *Nfil3* at 20 weeks of age (see Supplementary material online, *Table S16*). In addition, *Nfil3* was a hub gene in a co-expression module (purple; Supplementary material online, *Table S3*) that also contained the greatest number of candidate genes (*Grhl1*, *Ammecr1l*, *Hs6st1*, *Nfil3*, *Fam221a*, *Lmo4*, *Adamts1*).

NFIL3 (nuclear factor, interleukin 3 regulated, also known as E4BP4) is a TF involved in the control of circadian rhythm that is associated with BP and has links to the RAS. Circadian clock genes have been identified as abnormally expressed in the kidneys of SHR,^47^ and *Nfil3* is over-expressed in SHR adrenal glands.^48^ Conversely, *Nfil3* knockout mice have reduced systemic BP.^49^ In terms of interaction with the RAS, we observed significant reduction in *Nfil3* expression during RAS inhibition. Previous studies have demonstrated that NFIL3 has potent effects to increase expression of aldosterone synthase and aldosterone secretion, an effect that is amplified by angiotensin.^50^ Furthermore, *Nfil3* knockout results in the loss of angiopoietin-2,^49,51^ while the expression of angiopoietin-2 is reduced by angiotensin receptor antagonism.^52^ Therefore, reduced *Nfil3* expression as a result of RAS inhibition could further amplify the suppression of the RAS. Interestingly, recent genome-wide meta-analysis also identified *Nfil3* as significant potential gene target for hypertension.^53^

Of the other candidates we identified, ADAM metallopeptidase with thrombospondin type 1 motif, 1 (*Adamts1*) also showed significantly reduced renal expression at 20 weeks of age. ADAMTS1 is a metalloproteinase with a number of functions that include inhibition of angiogenesis,^54^ blood vessel remodelling,^55^ and the normal development of the kidney.^56^ Importantly, angiotensin II induces the expression of *Adamts1* in endothelial and vascular smooth muscle cells (VSMC).^57^ Mice with a genetic haploinsufficiency of *Adamts1* have significantly lower BP than controls.^58^

In contrast, the expression of *Bcl6* was significantly increased in the 20-week LOS SHR and showed the strongest negative correlation with *Ren* expression at that time. BCL6 is a sequence-specific transcriptional repressor that shows increased renal expression in young SHR^59^ but lower expression as hypertension develops in adulthood,^60^ as we also observed in vehicle SHR (*Figure 3*). Other studies have shown that over-expression of *Bcl6* significantly reduces SHR BP and reduces renal inflammation.^60^ At a mechanistic level, BCL6 can inhibit angiotensin II-induced proliferation of human VSMC and can attenuate angiotensin II-induced oxidative damage in VSMC and human renal tubular epithelial cells.^60^

These observations raise the potential role of inflammatory kidney damage in causing or sustaining hypertension in SHR.^61^ Such inflammation is believed to be stimulated by the activation of the RAS, and studies have shown that brief losartan treatment results in reduced urinary excretion of inflammatory markers.^61^ Reduced renal inflammatory damage may be a component of the legacy effect of RAS inhibition in SHR.

Because of their regulatory effects on gene expression,^23^ we specifically investigated miRNA expression and identified 45 miRNA molecules that were significantly differentially expressed at 20 weeks following losartan treatment (*Table 2*). Among these, the miRNA miR-145-3p^62^ was strongly negatively correlated (*r* < −0.8) with 9 out of the 13 candidate mRNA genes. miRNA-145 has been shown to be associated with SHR hypertension,^63,64^ and our observations suggest that it might play a significant role in the legacy effects following RAS inhibition.

To help understand how the identified candidate genes might influence the RAS, our multivariate analyses showed that seven of the candidate genes were highly correlated (|*r*| ≥ 0.8) with *Ren* at 20 weeks of age (see Supplementary material online, *Table S13*). In relation to miRNAs that might be involved in the suppression of Ren expression, miR-145-3p showed the highest negative correlation (*r* = −0.77; Supplementary material online, *Table S15*). We also found links between lncRNA and miRNA expression at 20 weeks of age. In particular, there was a strong correlation (*r* = 0.92) between miRNA-145-3p and the lncRNA AC115371.3 (see Supplementary material online, *Table S11*). This lncRNA showed the greatest number of correlations with the 45 top differentially expressed miRNAs (see Supplementary material online, *Table S11*), and it was also highly negatively correlated (*r* = −0.82) with *Ren* expression (see Supplementary material online, *Table S10*).

The origins of the gene network changes are likely during the active treatment period, where the key seems to be RAS inhibition, in addition to lowering BP. We demonstrated major perturbations in the expression of elements of the RAS during active RAS inhibitor treatment, including a six-fold increase in *Ren* expression with increased expression of angiotensinogen, angiotensin II receptor–associated protein, and transforming growth factor beta 1. Such RAS gene over-expression may evoke compensatory homeostatic responses at a molecular level that have the effect of resetting genomic networks that impact on the RAS and the kidney, leading to reduced *Ren* expression. It is worth noting that long-term reduction in SHR BP depends on a sufficient period of treatment and is most effective in younger animals,^2^ suggesting that duration and timing of treatment influence the resetting set-points for *Ren* and cognate gene expression.

An important question is why gene network changes should persist after treatment stops. Epigenetic factors such as DNA methylation, histone modification, and chromatin remodelling are relevant here. DNA methylation of CpG islands that are commonly found in promoter regions of genes^65^ characteristically occurs in a tissue-specific manner and can result in long-term effects by recruiting proteins involved in gene repression or by inhibiting the binding of TF(s) to DNA. In this regard, our finding of increased methylation in the promoter region of *Ren* suggests one likely explanation for long-term reduced *Ren* expression.

Towards understanding the legacy of RAS inhibition, we also sought to find clues as to why the SHR (and SHRSP) might be genetically predisposed to this effect. The likely explanation is differences in DNA sequence variation, and we focused on *Ren*. Based on the most comprehensive and accurate sequences available,^28,29^ we identified 112 variants in and around *Ren* that were found in SHR and SHRSP but not WKY or BN strains. Further experiments will be required to determine the potential role these variants may play in predisposition to the legacy effects of RAS inhibition. Of highest priority would be variants that may affect gene expression. In this respect, we identified numerous variants in putative binding motifs for TFs, many of which have been implicated previously in aspects of control of *Ren* expression.^37–40,43,66^ The impact of variants in the lncRNA LOC102550525 (antisense to *Ren*) also merits special attention given their diverse roles in regulation of gene expression.^67^

The overall picture emerging from these analyses is that the genomic landscape of renal expression during the legacy phase after RAS inhibition is a network of changes that involve the RAS itself. It would seem reasonable to attribute the long-term BP reduction to the lower renal *Ren* expression, particularly given that the kidneys are the primary source of renin and renin is the rate-limiting factor in the RAS. It might also explain why the legacy BP effect follows the kidney in renal transplantation experiments. However, within the network of DE that we have been able to resolve, at this stage, it is not possible to know the sequence of events or the hierarchy of control that results in the reduced *Ren* expression and persistently lower BP or indeed whether certain candidates themselves contribute directly to the lower BP. Further experiments are needed to test the specific contributions of individual candidates that elucidate their functional molecular interrelationships. Although, the SHR has long been considered a highly relevant model of essential hypertension, the direct significance of these experiments to humans remains uncertain. A more detailed knowledge of the mechanisms in SHR should help clarify the potential to prevent human hypertension and inform future clinical trials.

**Translational perspective**

These experiments provide the foundation for a more thorough understanding of the molecular basis of the prevention of genetic hypertension in SHR. There is potential for future strategies using novel therapeutic targets to achieve long-lasting effects on BP. Such approaches might be incorporated in further testing of the prevention paradigm in humans.

## Supplementary Material

cvae053_Supplementary_Data

## Data Availability

We have provided the full outputs from all main analyses in the Supplementary files.
