# Supplementary material for: Four-week inhibition of the renin–angiotensin system in spontaneously hypertensive rats results in persistently lower blood pressure with reduced kidney renin and changes in expression of relevant gene networks
Source: Cardiovasc Res. 2024 Mar 19;120(7):769–81. doi: 10.1093/cvr/cvae053 (PMC11135646; doi:10.1093/cvr/cvae053)
Supplement: cvae053_Supplementary_Data [file cvae053_supplementary_data.zip › Revised Supp Methods .docx]

**Supplementary Materials and Methods:**

**Methods**

**Telemetric direct arterial pressure recordings**

Long-term continuous recordings of mean arterial pressure (MAP) and heart rate (HR) in conscious state were performed using a radiotelemetry system (DSI, St.Paul, MN, USA). Briefly, the rats were lightly anesthetised by inhalation of 2% isoflurane (ISOFLOTM, Zoetis, Australia) in an induction box prior to intramuscular injection of a mixture of ketamine (60 mg/kg, i.m.; Lyppard, Dingley, Australia) and medetomidine (250 µg/kg, i.m.; Pfizer Animal Health, West Ryde, Australia). Body temperature was maintained throughout and he adequate depth of anesthesia was monitored by respiratory and heart rate. Analgesia was provided by injection of a non-steroidal, anti-inflammatory agent (meloxicam, 1 mg/kg, s.c., Metacam, Boehringer Ingelheim, Sydney, Australia). The surgery started after loss of pedal withdrawal and corneal reflexes indicating the required deep surgical level of anesthesia. The eye’s moisture was maintained by gel application (Polyvisc). The surgical field was shaved and disinfected with 80% ethanol, chlorhexidine and betadine antiseptic solution. An incision in the midline of the abdominal cavity was made to enable isolation of the abdominal aorta caudal to the renal arteries. The catheter of the telemetry transmitter (TA11PAC40, DSI, St.Paul, MN, USA) was inserted into the abdominal aorta following the manufacturer’s instructions. At the end of the surgery, the animals were injected with warmed Hartmann’s solution (1 mL, i.p.; Baxter Healthcare, Australia), and the anesthesia was reversed with atipamazole (1 mg/kg, i.m.; Pfizer). Rats were placed on a heat mat for recovery, and then returned to their standard cage. The animals were monitored daily and allowed to completely recover for 10 days, before the recordings started. Both body weight assessment and 24 h BP recordings were performed once a week in each rat for 11 weeks. The BP recordings were sampled in 10 s segments with an acquisition frequency of 1000 Hz. The data summarized in the manuscript are an average from a 2h recording period during the dark (between 3:45-5:45 am) period.

**Tail-cuff blood pressures**

We also measured tail cuff systolic blood pressure (SBP) in larger groups of animals. Animals were accustomed to the light restraint and BP measurement protocol and SBP was measured using a programmed sphygmomanometer with a pneumatic pulse transducer (PE-300; Narco Bio-System, Houston, TX).

**Tissue collection and RNA extraction**

Animals were rapidly euthanized using 2% isoflurane and ketamine (100 mg/kg, i.m.; Lyppard, Dingley, Australia) and the kidneys were dissected on ice to obtain cortical tissue. All tissues were immediately submerged in RNA*later* stabilisation solution (Thermo Fisher Scientific), frozen in liquid nitrogen and stored at -80°C. Total RNA was extracted from dissected renal cortices from SHR vehicle (VEH), losartan (LOS) and hydralazine (HYD) treated animals using the miRVana™ miRNA isolation kit (Thermo Fisher Scientific) according to manufacturer’s instructions.

**Total RNA and miRNA sequencing, read processing and differential gene expression**

Total RNA-sequencing, specific miRNA and methylation sequencing was performed by the Australian Genome Research Facility (Melbourne, Australia). Total RNA sequencing was also obtained from Novogene (Beijing, China) for certain groups including confirmation of VEH and LOS differential expression results. A total of 3 sequencing runs by AGRF and Novogene were made to accommodate all samples and differential expression analyses were made only by comparison within individual runs to avoid potential batch effects. RNA-sequencing was performed on samples from n=9 VEH (n=5 at 14 weeks and n=4 at 20 weeks of age), n=10 LOS (n=5 at 14 and n=5 at 20 weeks of age), n=3 HYD (20 weeks of age), n=5 PER (20 weeks of age).

All total RNA analyses utilized the same parameters including rRNA removal (Ribo-Zero depletion) and Illumina HiSeq 150bp paired-end sequencing at high depth (~100 million reads per sample). Read quality was assessed using the FastQC software version 0.11.8 ([www.bioinformatics.babraham.ac.uk/projects/fastqc/](http://www.bioinformatics.babraham.ac.uk/projects/fastqc/)) and samples all showed highly quality base scores. Alignment and quantification of total RNA-sequencing data was performed using Rsubread aligner (version 1.34.6). Paired-end 150bp Illumina reads were aligned to the rat genome (UCSC rn6 assembly) at the gene level with an average of 97.6% (SD 0.85%) of reads successfully mapped. Genes with greater than one count-per-million mapped reads in at least two samples were retained for further analysis, genes below this threshold were filtered out.

miRNA single-end 50bp Illumina reads were quality checked using FastQC version 0.11.8 ([www.bioinformatics.babraham.ac.uk/projects/fastqc/](http://www.bioinformatics.babraham.ac.uk/projects/fastqc/)) and samples all showed highly quality base scores. miRNA reads were aligned and quantified with Oasis 2.0,^1^ an online software package specialised for small RNA-sequencing data including trimming of the Illumina adapter sequence from fastq files, read filtering (15–32 nt) and removal of low abundance reads (< 5 reads). Remaining reads were mapped to the rat genome rn6 and miRBase v22 with an average of 75.6% (±10.2 SD) of reads successfully mapped.

Analysis of differential expression in total RNA and miRNA sequencing data was performed in the R statistical programming environment (version 3.5.2) using edgeR (version 3.26.7), EDAseq (version 2.18.0) and RUVseq (version 1.18.0) Bioconductor packages. Biological coefficient of variation (BCV) was checked using the common dispersion method (negative binomial dispersion by conditional maximum likelihood). BCV is derived by subtracting the estimated technical variation (i.e. measurement error) from total CV across libraries (see edgeR manual available on Bioconductor.^2^

Data normalisation including adjusting for library size and removal of potential batch/technical effects was performed using EDAseq and RUVseq (Remove Unwanted Variation from RNA-Seq data). The function betweenLaneNormalisation in EDAseq was used for sequencing depth normalisation among samples using a nonlinear full quantile method.^3^ A second normalisation step based on factor analysis of putative non-differentially expressed genes was performed. This included producing a set of *in silico* empirical control genes (undifferentiated FDR q > 0.95) via differential expression analysis in edgeR.^4^ These negative control genes were used in the RUVg normalising function in RUVseq,^2^ removing k=2 factors of unwanted variation. Normalization success was checked by plotting the relative log expression (plotRLE function) and the PCA (plotPCA function) across samples using EDAseq.

Differential expression analysis was performed in edgeR, which applies a trimmed mean of M values (TMM) normalisation where dispersion parameters for each gene are estimated with the Cox-Reid common dispersion method^5^ and employed in a negative binomial generalized linear model for each gene. Accounting for gene dispersion ensures that expression differences that are consistent between replicates are more highly weighted than those that are not to ensure differential expression is not driven by outliers. P-values were adjusted for multiple testing using the Benjamini-Hochberg correction with an FDR q-value < 0.05.

**Methylation sequencing and read processing**

Illumina NovaSeq (50bp single-end reads, ~10-20 million reads per sample) was used for methylation sequencing using the reduced representation bisulfite sequencing (RRBS) technique. RRBS single-end 100bp Illumina reads were processed using the following steps also described in the protocol by Chen *et al*.^6^ and the edgeR package user’s manual.^7^ Reads were first trimmed with Trim Galore version 0.6.6,^8^ a wrapper for cutadapt version 3.5,^9^ with the *-rrbs* flag set to remove adapters and trim poor quality reads. Trimmed reads were then aligned with Bismark version 0.22.3,^10^ to the rn6 rat genome using Bowtie2. Methylation calls were made using the bismark_methylation_extraction function and read into R with the readBismark2DGE function in edgeR. CpGs on unassembled chromosomes and those assigned to the Y chromosome and mitochondrial DNA were removed, and CpGs were annotated with the identity of the nearest gene with the nearest transcription start site (TSS) function. CpGs with low coverage were filtered out by summing the counts of methylated and unmethylated reads to get the total read coverage at each CpG site for each sample. CpGs with a total count (methylated and unmethylated) of less than 8 in every sample were removed as well as CpGs that were never methylated or always methylated as they provide no information about differential methylation^5^.

**Genome wide differential methylation analyses for CpGs and gene promoters**

The genome-wide analyses detected over 9 million CpGs from which we filtered out CpG loci with very low reads across samples. Testing for differential methylation was performed using the ratio between methylated and unmethylated counts modelled by a negative binomial linear model in edgeR. To assess methylation in gene promoters, CpG counts were aggregated 2kb upstream to 1kb downstream of the transcription start site (TSS) for all annotated genes. The gene promoter approach fits well with the observation that CpG methylation in promoter regions is often associated with silencing of transcription and gene expression.^11^ To assess methylation in CpG clusters, CpG counts were aggregated in naturally occurring CpG-rich islands (see methods for aggregation in section below). We did not interpret methylation at individual CpGs (i.e. at the highest possible resolution of methylation) due to variation in methylation status (methylated/unmethylated) per CpG across samples and differences in differential methylation often observed between directly adjacent CpGs (within islands) which often resulted from low/variable read numbers per CpG even after applying standard read count filters in edgeR.

**Genome wide differential methylation analysis for CpG clusters**

In order to investigate patterns of genome wide methylation outside of gene promoter regions, we assessed methylation in CpG clusters (islands) with custom R code. This code was designed to scan across the entire genome and aggregate counts (same as applied in the gene promoter analysis) into naturally occurring CpG clusters – every time a gap between two CpGs of ≥500bp was encountered, a new CpG cluster was assigned. This was initially tested on a smaller subset of genes and matched well with CpGs that visually clustered within those genes. This genome-wide cluster-level approach fits with the finding that over half of all CpG islands are found outside of TSS/promoter regions.^12^

**Multivariate analyses in MixOmics to explore potential gene co-regulation between coding and non-coding genes at 20 weeks related to Losartan treatment**

Given the importance of gene regulatory functions of non-coding RNA, we investigated multivariate correlations between differentially expressed (FDR q<0.05) coding and non-coding RNA in all datasets that included: 272 protein coding genes, 15 lincRNAs and 19 other non-coding RNAs identified from the total RNA-seq datasets (see Table S1); 45 miRNAs identified in the miRNA-seq dataset (see Table S2).

We used a multivariate framework which allows expression of all differentially-expressed coding and non-coding genes across LOS and VEH samples to be compared (correlated) simultaneously, while also employing hierarchical clustering (complete linkage) to group coding and non-coding genes with highly similar gene expression profiles which can be used to infer potential gene co-regulation. We employed regularized Canonical Correlation Analysis (rcc function) in the R package mixOmics version 6.6.2.^13^ to perform the multivariate correlations. These multivariate correlation patterns were then visualised with clustered image maps (cim function, mixOmics) from which network diagrams were constructed with only the most highly correlated (|r| ≥ 0.8) genes to form hypotheses about potential co-regulatory relationships between coding and non-coding genes.

*Coding vs non-coding differentially expressed genes:* The clustered image map in Figure S2A shows all differentially expressed coding and non-coding genes and provides a broad overview of gene clustering within and between coding and non-coding genes. Figure S2B which was derived from Figure S2A shows only coding and non-coding genes that were highly correlated (|r| ≥ 0.8) only – this network diagram was constructed in Cytoscape version 3.7.1. Figure S2C shows a simplified network diagram with only the 13 candidate genes.

*Co-regulation between RAS genes and 35 confirmed mRNAs and 45 miRNAs:* To explore potential co-regulation between RAS genes and the 35 confirmed genes at 14 and 20 weeks, as well as between RAS genes and miRNAs at 20 weeks, we performed multivariate analyses (same methods described above) between each dataset. Figure S5 (A-C) shows the clustered image maps and derived highly correlated (|r| ≥ 0.8) pairs only, shown in the network diagrams.

**Co-expression analyses in WGCNA to define gene networks at 20 weeks related to Losartan treatment and BP differences**

We used WGCNA version 1.68,^14^ which aims to build a gene networks which are based on sets of modules consisting of highly interconnected genes with similar expression profiles. These can be used to analyse the internal relationship between key genes in modules and clinical characteristics. The main analysis steps are as follows: a correlation matrix between pairs of expressed genes is generated; hierarchical clustering is performed before constructing the co-expression network, and a soft thresholding power (β) is used to conform the relationship between genes in the gene co-expression network to the scale-free network distribution; the adjacency matrix is then converted into a topological overlap matrix (TOM), and the corresponding degree of difference (1-TOM) calculated; a minimum number of genes per module is defined and modules divided according to the standard of a dynamic shear tree; finally, module statistics (e.g. module eigengenes, module membership) are calculated and modules can be directly correlated with clinical characteristics.^15^

Three WGCNA networks were generated, one for each class of studied genes (mRNAs, miRNAs and non-protein coding RNAs such as lncRNAs and snoRNAs). In order to retain a sufficient number of genes for network construction in WGCNA yet still focus on genes related to treatment, we included any differentially expressed gene with a nominal p-value<0.05 from the total RNA-seq data (n=2844 protein coding, Table S3; 186 non-coding RNAs, Table S4). For the miRNA data, we chose a more liberal p threshold (p<0.10) to retain a sufficient number of genes for network construction (n=210 miRNAs, Table S5).

For each network, genes within modules were assessed for their intramodular connectivity (IC, connectivity of genes with other genes within the same module), module membership (MM, correlation of individual gene expression with module eigengene (ME) of its respective module) and hub genes. In scale-free network topology, ‘hubs’ are the most highly connected genes (of which there are typically relatively few) that correspond with high values for IC and MM. To identify gene modules (coding and non-coding) that might be related to the legacy effect on BP, we examined the correlation between ME and SBP (Tables S3-S5).

**Gene ontology enrichment analysis to examine biological pathways related to Losartan treatments effects and the RAS**

To obtain an insights into potential functional relevance of differentially-expressed genes we undertook two gene ontology (GO) enrichment analyses with the Panther Classification System release 17.0 in the Gene Ontology (release 2023-05-10) application.^16,17^ Analysis 1 included mRNA genes in modules (Table S3) that had been defined from WGCNA analyses, however there were often insufficient numbers of genes within modules to perform the overrepresentation tests in the GO analysis. Analysis 2 included leading differentially expressed genes related to LOS treatment (n=1676, Table S7). These analyses accounted for multiple testing (FDR) by applying the Benjamini-Hochberg procedure to Fisher’s exact test p-values.

**Renin immunolabelling**

Immunohistochemistry for renin was performed as described previously^18,19^ and all analyses were blinded to the treatment groups. Briefly, rat kidneys were fixed in 10% buffered formalin. Five μm paraffin sections of kidney were incubated with normal goat serum for 1 hour (5425S, Cell Signaling Technology), and then overnight at 4°C with a polyclonal mouse renin protein antibody raised against pure mouse submandibular gland renin (1:8000) (1. 2). A negative control without the primary antibody was included. Sections were washed with 0.1M phosphate buffered saline (PBS), incubated for 1 hour with biotin-conjugated goat anti-rabbit IgG (1:500, 111-065-144, Jackson ImmunoResearch, Pennsylvania, USA), washed with PBS and then incubated with the Vectastain ABC standard kit (Vector Laboratories, Pennsylvania, USA) for 30 minutes and liquid DAB substrate chromogen kit (BD Pharmingen, BD Biosciences, CA, USA) for 5 minutes. Following rinsing with tap water, sections were counterstained in Mayer's hematoxylin and coverslipped. For quantitation, four randomly chosen sections from each kidney at least 125 μm apart were selected. Images of renin immunolabelling associated with the juxtaglomerular apparatus (JGA) from 50 glomeruli per section were captured at 100X magnification using a digital microscope camera (DS-Ri2, Nikon, Japan) attached to an upright microscope (H550L, Nikon, Japan. The fraction of JGAs that were renin positive was averaged across four sections from each sample.

1. Capece V, Garcia Vizcaino JC, Vidal R, Rahman RU, Pena Centeno T, Shomroni O, Suberviola I, Fischer A, Bonn S. Oasis: online analysis of small RNA deep sequencing data. *Bioinformatics*. 2015;**31**:2205-2207.

2. https://bioconductor.org/packages/release/bioc/html/edgeR.html.

3. Risso D, Schwartz K, Sherlock G, Dudoit S: GC-content normalization for RNA-Seq data. *BMC Bioinformatics*. 2011,**12**:480.

4. Robinson MD, McCarthy DJ, Smyth GK. edgeR: a Bioconductor package for differential expression analysis of digital gene expression data. *Bioinformatics*. 2010;**26**:139-140.

5. McCarthy DJ, Chen Y, Smyth GK. Differential expression analysis of multifactor RNA-Seq experiments with respect to biological variation. *Nucleic Acids Res*. 2012;**40**:4288-4297.

6. Chen Y, Pa, B, Visvader JE, Smyth GK. Differential methylation analysis of reduced representation bisulfite sequencing experiments using edgeR. *F1000Research*. 2017;**6**:2055.

7. Chen Y, McCarthy D, Ritchie M, Robinson M, Smyth G. edgeR: differential analysis of sequence read count data. User’s Guide Version 3.42.4 (June 4, 2023).

8. https://www.bioinformatics.babraham.ac.uk/projects/trim_galore/.

9. Martin M. Cutadapt removes adapter sequences from high-throughput sequencing reads.*EMBnet.journal*. 2011;**17**:10-12.

10. https://www.bioinformatics.babraham.ac.uk/projects/bismark.

11.Bird AP. CpG-rich islands and the function of DNA methylation. *Nature*. 1986;**321**: 209-213.

12. Cain JA, Montibus B, Oakey RJ. Intragenic CpG islands and their impact on gene regulation. *Front Cell Dev Biol*. 2022;**10**:832348.

13. Rohart F, Gautier B, Singh A, Lê Cao KA. mixOmics: An R package for 'omics feature selection and multiple data integration. *PLoS Comput Biol*. 2017;**13**:e1005752.

14. Langfelder P, Horvat, S. WGCNA: an R package for weighted correlation network analysis. *BMC Bioinformatics*. 2008;**9**:559.

15. Zhang B, Horvath S. A general framework for weighted gene co-expression network analysis. *Stat Appl Genet Mol Biol*. 2005;**4**:Article17.

16. Ashburner M, Ball CA, Blake JA, Botstein D, Butler H, Cherry JM, Davis AP, Dolinski K, Dwight SS, Eppig JT, Harris MA, Hill DP, Issel-Tarver L, Kasarskis A, Lewis S, Matese JC, Richardson JE, Ringwald M, Rubin GM, Sherlock G. Gene ontology: tool for the unification of biology. The Gene Ontology Consortium. *Nat Genet*. 2000;**25**:25-29.

17. Mi H, Huang X, Muruganujan A, Tang H, Mills C, Kang D, Thomas PD. PANTHER version 14: more genomes, a new PANTHER GO-slim and improvements in enrichment analysis tools. *Nucleic Acids Res*. 2019;**47**:D419-D426.

18. Berka JLA, Alcorn D, Ryan G, Skinner SL. Renin processing studied by immunogold localization of protein and renin in granular juxtaglomerular cells in mice treated with enalapril. *Cell Tissue Res*. 1992;**268**:141-148.

19. Kelly DJ, Skinner SL, Gilbert RE, Cox AJ, Cooper MR, Wilkinson-Berka JL. Effects of endothelin or angiotensin II receptor blockade on diabetes in the transgenic (mRen-2)27 rats. *Kidney Int*. 2000;**57**:1882-1894.
