# Supplementary material for: Four-week inhibition of the renin–angiotensin system in spontaneously hypertensive rats results in persistently lower blood pressure with reduced kidney renin and changes in expression of relevant gene networks
Source: Cardiovasc Res. 2024 Mar 19;120(7):769–81. doi: 10.1093/cvr/cvae053 (PMC11135646; doi:10.1093/cvr/cvae053)
Supplement: cvae053_Supplementary_Data [file cvae053_supplementary_data.zip › Supplementary Figures.docx]

**SUPPLEMENTARY FIGURES**

**Figure S1: Comparison of log base 2 fold change (log_2_FC) expression for 35 genes between LOS and VEH SHR animals at 20 weeks.** X-axis represents the initial (main AGRF) dataset, y-axis represents the validation (Novogene) dataset.

**Figure S2: Multivariate correlations between differentially expressed coding and non-coding genes.** (A) Clustered Image Map from canonical correlation analysis in MixOmics showing all correlations (see Table S10) and clustering patterns between coding (y-axis, not all gene labels shown) and non-coding (x-axis, not all gene labels shown) genes based on hierarchical clustering. (B) Cytoscape network between most highly correlated (|r| ≥ 0.8) coding and non-coding genes. Symbol size is determined by number of correlations (|r| ≥ 0.8) between coding and non-coding genes. Line weight determined by magnitude of correlation with negative correlations emphasized (Cytoscape edge width: r = -0.8 to -1.0 with widths 0.1 to 3 respectively; r = 0.8 to 1.0 with widths 0.05 to 1 respectively).


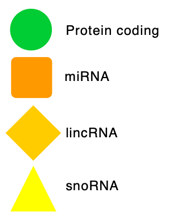


(B)

(A)

(C) Simplified network map (derived from Fig S2A-B) with only the 13 candidate genes (Table 1A) shown. Cytoscape network (right) shows only most highly correlated (|r| ≥ 0.8) genes. Two candidate (*Nfil3*, *Cish*) genes are not shown as they were not highly correlated (|r| < 0.8) with any non-coding genes. Network line weight determined by magnitude of correlation with negative correlations emphasized (Cytoscape edge width: r = -0.8 to -1.0 with widths 0.1 to 3 respectively; r = 0.8 to 1.0 with widths 0.05 to 1 respectively).

**Figure S3: Multivariate correlations between differentially expressed lncRNAs, miRNAs and Ren.** Clustered Image Map (left) from canonical correlation analysis in MixOmics showing all correlations and clustering patterns between miRNAs (y-axis, not all gene labels shown), Ren and lncRNA (x-axis) genes based on hierarchical clustering. Cytoscape network (right) between most highly correlated (|r| ≥ 0.8) genes. Symbol size is determined by number of correlations (|r| ≥ 0.8) between genes. Line weight determined by magnitude of correlation with negative correlations emphasized (Cytoscape edge width: r = -0.8 to -1.0 with widths 0.1 to 3 respectively; r = 0.8 to 1.0 with widths 0.05 to 1 respectively).

**Figure S4. WGCNA analysis for mRNA (A), ncRNA (B) and miRNA (C).** Left panel shows cluster dendrogram and module assignment, right panel includes correlation (upper values, vertical scale bar) and associated p-values (lower values) between module eigengene and systolic blood pressure measured at 20 weeks

(A) mRNA

(B) ncRNA

(C) miRNA

**Figure S5.** Clustered image maps and network diagrams based on correlations for RAS genes (|r| ≥ 0.8). (A) correlations at 20 weeks with validated 35 mRNA for differentially expressed genes identified at 20 weeks of age, (B) correlations at 14 weeks with validated 35 mRNA for differentially expressed genes identified at 20 weeks of age and (C) correlations at 20 weeks with top 45 differentially expressed miRNA identified at 20 weeks of age. Network line weight determined by magnitude of correlation with negative correlations emphasized (Cytoscape edge width: r = -0.8 to -1.0 with widths 0.1 to 3 respectively; r = 0.8 to 1.0 with widths 0.05 to 1 respectively).

(A) mRNA 20 weeks

(B) mRNA 14 weeks

(C) miRNA 20 weeks
